# Health Tract, No. 154

**Published:** 1865

**Authors:** 


### HEALTH TRACT, No. 154.

### DISINFECTANTS.

The best disinfectants are those which cost the least, are most easily applied, and which cause the least inconvenience to the health, or the textures to which they are applied. If a disinfectant corrodes metals, stains garments, disfigures furniture, or is poisonous when outwardly applied or swallowed, it is comparatively valueless.

There is no disinfectant universally applicable. But it may be truly said that the best plan, and one which every clean, tidy, and sensible person would instinctively adopt, is to remove all causes of disagreeable or unhealthy odors ; disinfectants should only be used when that is impracticable. Many persons burn sugar in the sick-room ; this destroys nothing ; it is merely a deodorizer, all that was there before is still present, it is only giving a stronger odor ; it in reality only renders the air of the room more impure and more hurtful, not only to the one who is sick, but to every visitor and attendant ; in fact, the actual tendency is to diminish the chances of recovery. Besides, a disinfectant may destroy a special ill odor, but may be in itself more hurtful than the odor it was intended to obviate. From all the knowledge yet obtained on the subject, it does not appear that the odor of decaying animal substances is particularly injurious to the health, not even that which arises in the dissecting-room, or in the removal of the dead from burying-grounds, where the scent has been so stenchy as to cause fainting or an approach to suffocation, and the workmen had to be relieved every few minutes, no disease followed. Still, it is of curious interest to know that the odor escaping from human bodies, alive, sick, or well, will produce the most deadly forms of typhoid and ship-fever in a few hours. Hence, never use disinfectants until every possible effort at cleanliness has failed to secure a pure atmosphere.

Sinks, Privies, etc.—One pound of copperas, known as *sulphate of iron*, costing but a few cents, dissolved in four gallons of water, poured over a sink two or three times, will most completely destroy all offensive odors. Repeat during hot weather as needed. Musty Cellars are rectified in the same way, or by sprinkling the copperas itself over the floor, besides being beneficial in keeping rats away.

The *Scientific American* says : One pint of the liquor of chloride of zinc, in about two gallons of water, and one pound of chloride of lime, in two other gallons of water, then mixed, is perhaps the most effective of any thing that can be used ; and when thrown upon decayed vegetable matter of any description, will effectually destroy many offensive odors. Chloride of lime, or common quicklime, is better to scatter about damp places and heaps of filth.

Four parts of ground plaster of Paris, and one part of pulverized charcoal, well mixed, is an excellent absorbent of all noisome smells. The powdered charcoal alone applied to glass vessels and any table-ware, after being well washed with soap and water, effectually removes all odor. The best purifier of bad breath is to take a teaspoonful of finely pulverized charcoal in the mouth on going to bed ; it need not be swallowed, but simply allowed to remain around the teeth, gums, cheek, etc.

The hypochlorites, as well as the solutions of bromine and iodine, act admirably in destroying miasm and disinfecting the air. The manganate of soda or potash, dissolved in warm water and poured into sinks or drains, not only prevents the sending forth of disease, but gives out at the same time a considerable amount of oxygen to refresh the atmosphere.

